# Coronaviruses Lacking Exoribonuclease Activity Are Susceptible to Lethal Mutagenesis: Evidence for Proofreading and Potential Therapeutics

**DOI:** 10.1371/journal.ppat.1003565

**Published:** 2013-08-15

**Authors:** Everett Clinton Smith, Hervé Blanc, Marco Vignuzzi, Mark R. Denison

**Affiliations:** 1 Department of Pediatrics, Vanderbilt University Medical Center, Nashville, Tennessee, United States of America; 2 The Elizabeth B. Lamb Center for Pediatric Research, Vanderbilt University Medical Center, Nashville, Tennessee, United States of America; 3 Institut Pasteur, Centre National de la Recherche Scientifique Unité de Recherche Associée 3015, Paris, France; 4 Department of Pathology, Microbiology and Immunology, Vanderbilt University Medical Center, Nashville, Tennessee, United States of America; Washington University School of Medicine, United States of America

## Abstract

No therapeutics or vaccines currently exist for human coronaviruses (HCoVs). The Severe Acute Respiratory Syndrome-associated coronavirus (SARS-CoV) epidemic in 2002–2003, and the recent emergence of Middle East Respiratory Syndrome coronavirus (MERS-CoV) in April 2012, emphasize the high probability of future zoonotic HCoV emergence causing severe and lethal human disease. Additionally, the resistance of SARS-CoV to ribavirin (RBV) demonstrates the need to define new targets for inhibition of CoV replication. CoVs express a 3′-to-5′ exoribonuclease in nonstructural protein 14 (nsp14-ExoN) that is required for high-fidelity replication and is conserved across the CoV family. All genetic and biochemical data support the hypothesis that nsp14-ExoN has an RNA proofreading function. Thus, we hypothesized that ExoN is responsible for CoV resistance to RNA mutagens. We demonstrate that while wild-type (ExoN+) CoVs were resistant to RBV and 5-fluorouracil (5-FU), CoVs lacking ExoN activity (ExoN−) were up to 300-fold more sensitive. While the primary antiviral activity of RBV against CoVs was not mutagenesis, ExoN− CoVs treated with 5-FU demonstrated both enhanced sensitivity during multi-cycle replication, as well as decreased specific infectivity, consistent with 5-FU functioning as a mutagen. Comparison of full-genome next-generation sequencing of 5-FU treated SARS-CoV populations revealed a 16-fold increase in the number of mutations within the ExoN− population as compared to ExoN+. Ninety percent of these mutations represented A:G and U:C transitions, consistent with 5-FU incorporation during RNA synthesis. Together our results constitute direct evidence that CoV ExoN activity provides a critical proofreading function during virus replication. Furthermore, these studies identify ExoN as the first viral protein distinct from the RdRp that determines the sensitivity of RNA viruses to mutagens. Finally, our results show the importance of ExoN as a target for inhibition, and suggest that small-molecule inhibitors of ExoN activity could be potential pan-CoV therapeutics in combination with RBV or RNA mutagens.

## Author Summary

RNA viruses have high mutation rates (10^−3^ to 10^−5^ mutations/nucleotide/round of replication), allowing for rapid viral adaptation in response to selective pressure. While RNA viruses have long been considered unable to correct mistakes during replication, CoVs such as SARS-CoV and the recently emerged MERS-CoV are important exceptions to this paradigm. All CoVs encode an exoribonuclease activity in nonstructural protein 14 (nsp14-ExoN) that is proposed to prevent and/or remove misincorporated nucleotides. Because of the demonstrated resistance of SARS-CoV to the antiviral drug ribavirin (RBV), we hypothesized that ExoN is responsible for CoV resistance to RNA mutagens. Using RBV and the RNA mutagen 5-fluorouracil (5-FU), we show that CoVs lacking ExoN activity (ExoN−) are highly susceptible to RBV and 5-FU, in contrast to wild-type (ExoN+) CoVs. The inhibitory activity of 5-FU against ExoN− viruses resulted specifically from 5-FU incorporation during viral RNA synthesis that lead to extensive mutagenesis within the viral population, and was associated with a profound decrease in virus specific infectivity. These results demonstrate the proofreading activity of ExoN during virus replication and suggest that inhibitors of ExoN activity could be broadly useful inhibitors of CoV replication in combination with RBV or RNA mutagens.

## Introduction

The potential for CoVs to cause significant human disease is well demonstrated, with six known HCoVs—HKU1, OC43, NL63, 229E, SARS-CoV and MERS-CoV—causing colds, pneumonia, systemic infection, and severe or lethal disease [1]–[5]. Four of these viruses have been identified in just the last 10 years, with two, SARS-CoV and MERS-CoV, causing lethal respiratory and systemic infection [1], [3]–[6]. Studies over the past 10 years have expanded the known phylogenetic, geographic, and species diversity of CoVs, and support multiple emergence events of CoVs into humans from bats and other zoonotic pools [7]–[10]. The most recent evidence for CoV trans-species movement comes from the emergence of the novel MERS-CoV [1], [11], [12]. From April 2012 to June 2013 MERS-CoV has caused 72 laboratory confirmed cases and up to 50% mortality from severe respiratory and systemic disease in at least 8 countries, with evidence for human-to-human transmission [13]. MERS-CoV is most closely related to the bat CoVs HKU4 and HKU5 [11], and the recently identified receptor dipeptidyl peptidase 4 (DPP4) is present on both human and bat cells [14], providing a compelling argument that zoonotic CoV infections resulting in severe human disease may be more frequent events than previously thought. Because of the lack of epidemiological data, it remains unknown whether multiple introductions from a zoonotic source or human transmission of a mild or asymptomatic disease is responsible for these continuing cases of sporadic severe infections. However, based on the high mortality rates associated with SARS-CoV and those reported for MERS-CoV [13], this novel virus potentially represents a serious threat to global health for which no vaccines or therapeutics currently exist.

CoVs contain the largest known RNA genomes (27–32 kb) and encode an array of 16 viral replicase proteins, including a 3′-to-5′ exoribonuclease (ExoN) domain within nonstructural protein 14 (nsp14) [2], [15]–[17]. Similar to the proofreading subunit (ε) of *E. coli* DNA polymerase III, CoV nsp14-ExoN is a member of the DEDD superfamily of DNA and RNA exonucleases [15], [18]. This superfamily contains four conserved D-E-D-D acidic residues that are required for enzymatic activity, and mutation of these critical residues within CoV ExoN ablates or significantly reduces ExoN activity [15]. Studies from our group have demonstrated that ExoN activity is essential for high-fidelity replication in both the model CoV murine hepatitis virus (MHV) and SARS-CoV [19], [20]. Inactivation of ExoN activity due to alanine substitution of the first two active site residues results in 15- to 20-fold reduced replication fidelity in cell culture [19], [20] and a 12-fold reduction during SARS-CoV infection *in vivo* [21], associated with profound and stable attenuation of SARS-CoV virulence and replication. A recent study has shown that bacterially-expressed SARS-CoV nsp14-ExoN can remove mismatched nucleotides *in vitro*, and that ExoN activity is stimulated *in vitro* through interactions with the non-enzymatic CoV protein nsp10 [22]. Thus all bioinformatic, genetic and biochemical studies to date support the hypothesis that nsp14-ExoN is the first identified proofreading enzyme for an RNA virus and functions together with other CoV replicase proteins to perform the crucial role of maintaining CoV replication fidelity.

Retrospective clinical studies during the SARS epidemic ultimately concluded that treatment with ribavirin (RBV), an antiviral drug shown to be mutagenic for some RNA viruses [23], [24], was ineffective against SARS-CoV [25]–[28]. Because ExoN activity is required for CoV high-fidelity replication [19]–[21], we sought to determine if ExoN was responsible for CoV resistance to RNA mutagens. Using the nucleoside analog RBV and the base analog 5-fluorouracil (5-FU; [29]) we show that CoVs lacking ExoN activity (ExoN−) are up to 300-fold more sensitive to inhibition than wild-type CoVs (ExoN+). Additionally, using full-genome next-generation sequencing we show that ExoN− viruses accumulate 15- to 20-fold more A:G and U:C transitions, consistent with 5-FU incorporation during RNA synthesis. Ultimately our results suggest the exciting possibility that small-molecule inhibitors of ExoN activity could be potential pan-CoV therapeutics, especially when used in combination with RBV or RNA mutagens.

## Materials and Methods

### Cell culture and viruses

Murine astrocytoma delayed brain tumor cells (DBT cells) were grown at 37°C and maintained in DMEM (Invitrogen) containing 10% FBS, supplemented with penicillin, streptomycin, HEPES, and amphotericin B. VeroE6 (Vero) cells were grown at 37°C and maintained in MEM (Invitrogen) containing 10% FBS supplemented with penicillin, streptomycin, and amphotericin B. All work with MHV was performed using the reverse genetics infectious clone based on strain MHV-A59 [30], and work with SARS-CoV was performed using the reverse genetics infectious clone based on the Urbani strain [31]. Viral studies using SARS-CoV were performed in Select Agent certified BSL-3 laboratories using protocols reviewed and approved by the Institutional Biosafety Committee of Vanderbilt University and the Centers for Disease Control for the safe study and maintenance of SARS-CoV.

### Compounds and cell viability studies

5-fluorouracil (5-FU), ribavirin (RBV), guanosine (GUA) and mycophenolic acid (MPA) were obtained from Sigma. 5-FU and RBV were made as 200 mM stock solutions, and were prepared in DMSO and sterile water, respectively. GUA and MPA were prepared in DMSO as 40 mM or 100 mM stocks, respectively. Low concentration (µM) working stocks were prepared as needed in sterile water prior to dilution in DMEM. Viability of DBT and Vero cells was assessed using CellTiter-Glo (Promega) in 96-well plate format according to manufacturer's instructions. DBT and Vero cells were seeded into opaque tissue culture grade 96-well plates, and DMEM containing RBV or 5-FU was added to each well to achieve the concentrations indicated. Water or DMSO vehicle controls were performed, in addition to a 20% ethanol control for cell death. The cells were then incubated at 37°C for either 12 or 24 h, and cell viability was determined using a Veritas Microplate Luminometer (Promega). The resultant values were then normalized to untreated cells.

### Drug sensitivity studies and plaque assays

Subconfluent monolayers of DBT cells in 6-well plates were pretreated for 30 min at 37°C with 1 mL of DMEM containing vehicle or the indicated concentration of RBV, 5-FU, MPA, or GUA. The drug was then removed and cells were infected with MHV-ExoN+ or ExoN− viruses at an MOI of 1 plaque forming units (PFU)/cell (single-cycle) or 0.01 (multi-cycle) for 30 min at 37°C. Virus was then removed and 1 mL of DMEM containing vehicle, RBV, 5-FU, MPA, or GUA was added to each well. Cells were then incubated at 37°C for either 12 (single-cycle) or 24 (multi-cycle) h. The supernatant was harvested and virus titer was determined by plaque assay on DBT cells. For SARS-CoV studies, subconfluent monolayers of Vero cells in T25 flasks were pretreated for 30 min at 37°C with DMEM containing vehicle, RBV, or 5-FU. The drug was removed and cells were infected with either SARS-ExoN+ or ExoN− viruses at an MOI of 0.1 PFU/cell (single-cycle) for 30 min. The virus was removed and DMEM containing vehicle, RBV, or 5-FU was added back. Cells were then incubated for 24 h, at which point the supernatant was harvested and virus titer was determined by plaque assay on Vero cells. All treated samples were normalized to the untreated vehicle control, and values were expressed as fold change from untreated virus titers.

### Real-time quantitative reverse transcription PCR (real-time qRT-PCR) of viral genomic RNA

Viral RNA was harvested from infected cell monolayers using TRIzol reagent (Invitrogen), and was reverse transcribed (RT) using SuperScript III (Invitrogen). Random hexamers (1 µL of 50 µM stock) and 1 µg of total RNA were incubated for 5 min at 70°C. The remaining reagents were then added according to the manufacturer's protocol, and the mixture was incubated at 50°C for 1 h and then at 85°C for 5 min. All RT reactions were performed in a final volume of 20 µL. Real-time qRT-PCR was performed on the RT product using the Applied Biosciences 7500 Real-Time PCR System with Power SYBR Green PCR Master Mix (Life Technologies). Each reaction was performed in a total volume of 25 µL containing 12.5 µL of the Power SYBR Green PCR Master Mix, 125 ng each of the forward and reverse primers and 1 µL of the RT product which was diluted 1∶1000. Viral genomic RNA was detected using primers (forward: ACAGGGTGGAGTTCCCGTTA and reverse: ACGGAAGCACCACCATAAGA) optimized to generate a ∼120 nt portion of ORF1a. These values were normalized using the 2^−ΔΔCt^ method [32] to endogenous expression of the housekeeping gene glyceraldehyde-3- phosphate dehydrogenase (GAPDH) using primers (forward: GGGTGTGAACCACGAGAAAT and reverse: CCTTCCACAATGCCAAAGTT) optimized to yield a ∼120 nt portion of GAPDH [33], [34]. Triplicate wells of each sample were analyzed, and averaged into one value representing a single replicate to minimize well-to-well variation. The cycle parameters were as follows: Stage 1, (1 rep) at 50°C for 2 min; Stage 2, (1 rep) 95°C for 10 min; Stage 3, (40 reps) at 95°C for 15 sec and 57°C for 1 min. One representative product from each treatment was verified by melting curve analysis and agarose gel electrophoresis.

### Amplicon preparation for deep sequencing of whole viral genomes

Viral RNA from SARS-ExoN+ or ExoN− infected Vero monolayers was harvested using TRIzol reagent, and was reverse transcribed (RT) using SuperScript III as described above except with 5 µL of random hexamers (50 µM stock), 5 µg of total RNA, and in a final volume of 100 µL for each reaction. Four microliters of RT product was then used to generate 12 overlapping ∼3 kb amplicons for each virus treated with either 0 or 400 µM 5-FU by PCR. The high-fidelity polymerase Easy A (Agilent) was used to ensure that errors were minimal during PCR. All primer sets generated single bands which were then purified using the Wizard SV Gel and PCR Clean-Up System (Promega).

### Illumina next generation sequencing and analysis

Prior to sequencing, cDNA amplicons were fragmented (Fragmentase, NEB), clustered, and sequenced with Illumina cBot and GAIIX technology as previously described [35]. Between 1.4×10^8^ and 4.5×10^8^ bases, comprised of ∼69-nt reads, were obtained per virus, and CASAVA 1.8.2 was used to demultiplex and create the fastq files. Low quality bases from the ends of each sequence read were then trimmed, using *Phred* scores as the guiding metric (error probabilities higher than 0.001), and sequences with less than 16 bases after trimming were discarded to reduce false alignment and subsequent false variant calls. The program fastq-clipper (http://hannonlab.cshl.edu/fastx_toolkit/index. html) was used for this quality filtering. The Burrows-Wheeler Alignment tool was then used to align reads to the SARS-CoV ExoN+ or ExoN− reference genomes with a maximum of two mismatches per read [36]. Base calling at each position was determined using SAMTOOLS [37]. After the pileup, an in-house script collected the data per-position. For each position throughout the viral genome, the bases and their qualities were gathered, each variant allele's rate was initially modified according to its covering read qualities based on a maximum likelihood estimation and test for significance using Wilks' theorem. Additionally, an allele confidence interval was calculated and output for each allele. Only alleles with statistically significant p<0.05 values were retained and considered to be true variants. Above 0.01% all variants were found to be statistically significant, while below 0.01% many variants could not be distinguished from background error. Thus, the background noise caused by sequencing error was determined to be 0.01% or less.

### Statistical analysis

Statistical tests were applied where noted within the figure legends and were determined using GraphPad Prism (La Jolla, CA) software. Statistical significance is denoted (*P<0.05, **P<0.01, ***P<0.0001) and was determined using an unpaired, two-tailed Student's *t* test compared to either untreated samples or to the corresponding ExoN+ sample. For the cell viability studies, treated samples were compared to the DMEM sample containing DMSO.

## Results

### MHV-ExoN− viruses have increased sensitivity to RBV

**Figure 1:**
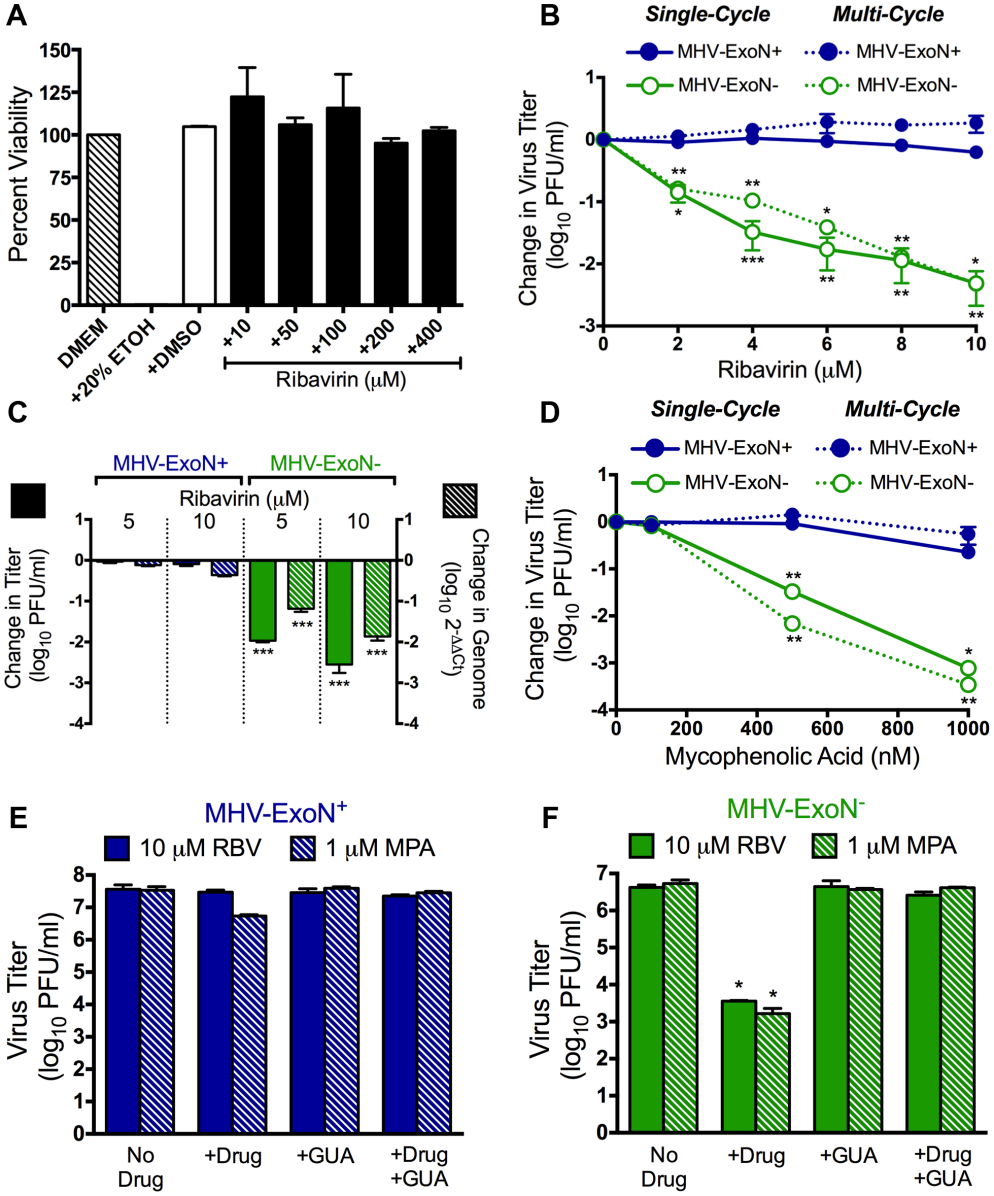
The antiviral activity of RBV against ExoN− viruses is not primarily due to mutagenesis. (**A**) DBT cells in 96-well plates were incubated with DMEM alone, or DMEM containing 20% ethanol (EtOH), 4% DMSO, or the indicated concentration of RBV for 12 h. Cell viability was determined using CellTiter-Glo (Promega) according to manufacturer's instructions. All values were normalized to the untreated (DMEM) control. No significant differences were found when RBV-treated values were compared to DMEM samples containing DMSO (+DMSO) using an unpaired, two-tailed Student's *t* test. Mean values ± S.E.M. are shown, n = 2. (**B**) MHV-ExoN+ (filled circle) and MHV-ExoN− (open circle) virus sensitivity to RBV during single- (solid lines; MOI = 1 PFU/cell) and multi-cycle (dotted lines; MOI = 0.01 PFU/cell) replication. MHV-ExoN+ viruses are shown in blue and MHV-ExoN− viruses are shown in green. The change in virus titer was calculated by dividing virus titers following treatment by the untreated controls. Mean values ± S.E.M. are shown, n = 4. (**C**) The change in titer (filled bars) and genomic RNA levels (hatched bars) of MHV-ExoN+ (blue) and MHV-ExoN− (green) viruses following treatment with RBV is shown. DBT cells were infected with MHV-ExoN+ or MHV-ExoN− in the presence or absence of RBV, and virus titer was determined by plaque assay. Genomic RNA levels were determined using two-step real-time qRT-PCR and primers optimized to amplify a ∼120 nt region of ORF1a [33]. The change in genomic RNA levels (2^−ΔΔCt^) is shown relative to endogenous GAPDH expression and was normalized to RNA levels from untreated samples. Mean values ± S.E.M. are shown, n = 6. (**D**) MHV-ExoN+ (filled circle) and MHV-ExoN− (open circle) virus sensitivity to mycophenolic acid (MPA) during single- (solid lines; MOI = 1 PFU/cell) and multi-cycle (dotted lines; MOI = 0.01 PFU/cell) replication. Mean values ± S.E.M. are shown, n = 2–4. RBV- or MPA-treated MHV-ExoN+ (**E**) and MHV-ExoN− (**F**) viruses with or without the addition of 100 µM guanosine (GUA) during single-cycle replication (MOI = 1 PFU/cell). Mean values ± S.E.M. are shown, n = 2. For all parts, statistical significance was determined using an unpaired, two-tailed Student's *t* test (*P<0.05, **P<0.01, ***P<0.0001).

Because RBV has been shown to be incorporated as ribavirin monophosphate (RMP) into viral RNA during replication [23], [24], [38]–[42], the presence of a proofreading enzyme would be predicted to exclude and/or remove nucleotide misincorporation [43]–[47]. If ExoN is responsible for the resistance phenotype, viruses lacking ExoN activity (ExoN−) should demonstrate increased titer reduction following RBV treatment as compared to wild-type viruses containing ExoN activity (ExoN+). To test this hypothesis, we examined the sensitivity of MHV-ExoN+ and ExoN− viruses to RBV during single-cycle (MOI = 1 PFU/cell) replication in murine astrocytoma delayed brain tumor cells (DBT cells). No toxicity was observed in DBT cells following treatment with up to 400 µM RBV (Figure 1A). MHV-ExoN+ viruses were resistant to 10 µM RBV (Figure 1B), while MHV-ExoN− virus titers decreased by ∼200-fold following treatment with 10 µM RBV. The capacity of 10 µM RBV to inhibit MHV-ExoN− replication is surprising because at least 10-fold higher concentrations of RBV are required to inhibit poliovirus and chikungunya viruses [48]–[50]. This observation could be due to the longer genomes of CoVs or to the mechanism(s) by which RBV inhibits CoV replication.

### The antiviral activity of RBV against ExoN− viruses is not primarily due to mutagenesis

If RBV is exerting antiviral activity primarily through mutagenesis following incorporation of RMP, MHV-ExoN− viruses should exhibit increased sensitivity during multi-cycle replication. To test this, we determined the sensitivity of MHV-ExoN+ and ExoN− viruses to RBV at a low multiplicity of infection (MOI = 0.01 PFU/cell). Unexpectedly, multi-cycle replication of MHV-ExoN− viruses in the presence of RBV (Figure 1B) was indistinguishable from single-cycle replication.

RBV has been reported to exert antiviral activity through numerous mechanisms [38] including disruption of viral RNA synthesis and inhibition of the cellular enzyme inosine monophosphate dehydrogenase (IMPDH). To determine if RBV treatment was affecting CoV RNA synthesis, we performed two-step real-time quantitative reverse transcription PCR (real-time qRT-PCR) to determine viral genomic RNA levels in the presence or absence of RBV. Similar to Figure 1B, MHV-ExoN+ titers were unaffected, whereas there was a dose-dependent reduction in MHV-ExoN− titers following RBV treatment (Figure 1C, filled bars). Corresponding dose-dependent reductions in MHV-ExoN− genomic RNA were observed (Figure 1C, hatched bars) following RBV treatment, demonstrating that treatment with 10 µM RBV decreased MHV-ExoN− RNA synthesis by nearly 100-fold during replication. Because RBV caused decreased RNA synthesis in MHV-ExoN− viruses, we calculated the relative specific infectivities of both viruses at each RBV concentration (Table 1). The relative specific infectivity of MHV-ExoN− viruses was decreased by 6- to 9-fold following treatment with RBV, while MHV-ExoN+ viruses were unaffected.

**Table 1:** Relative specific infectivities of MHV-ExoN+ and ExoN− viruses following treatment with RBV or 5-FU.

Virus	RBV(µM)	Relative Specific Infectivity	Fold Decrease	5-FU (µM)	Relative Specific Infectivity	Fold Decrease
MHV-ExoN+	0	1		0	1	
	5	1.2±0.1	0.84±0.06	100	0.33±0.05	3.4±0.5
	10	1.9±0.2	0.56±0.05	200	0.24±0.03	4.5±0.4
MHV-ExoN−	0	1		0	1	
	5	0.19±0.04	6.0±0.7^***^	100	0.10±0.03	13.6±2.9^**^
	10	0.26±0.11	9.1±3.0^*^	200	0.012±0.004	128±29^**^

**Figure 2:**
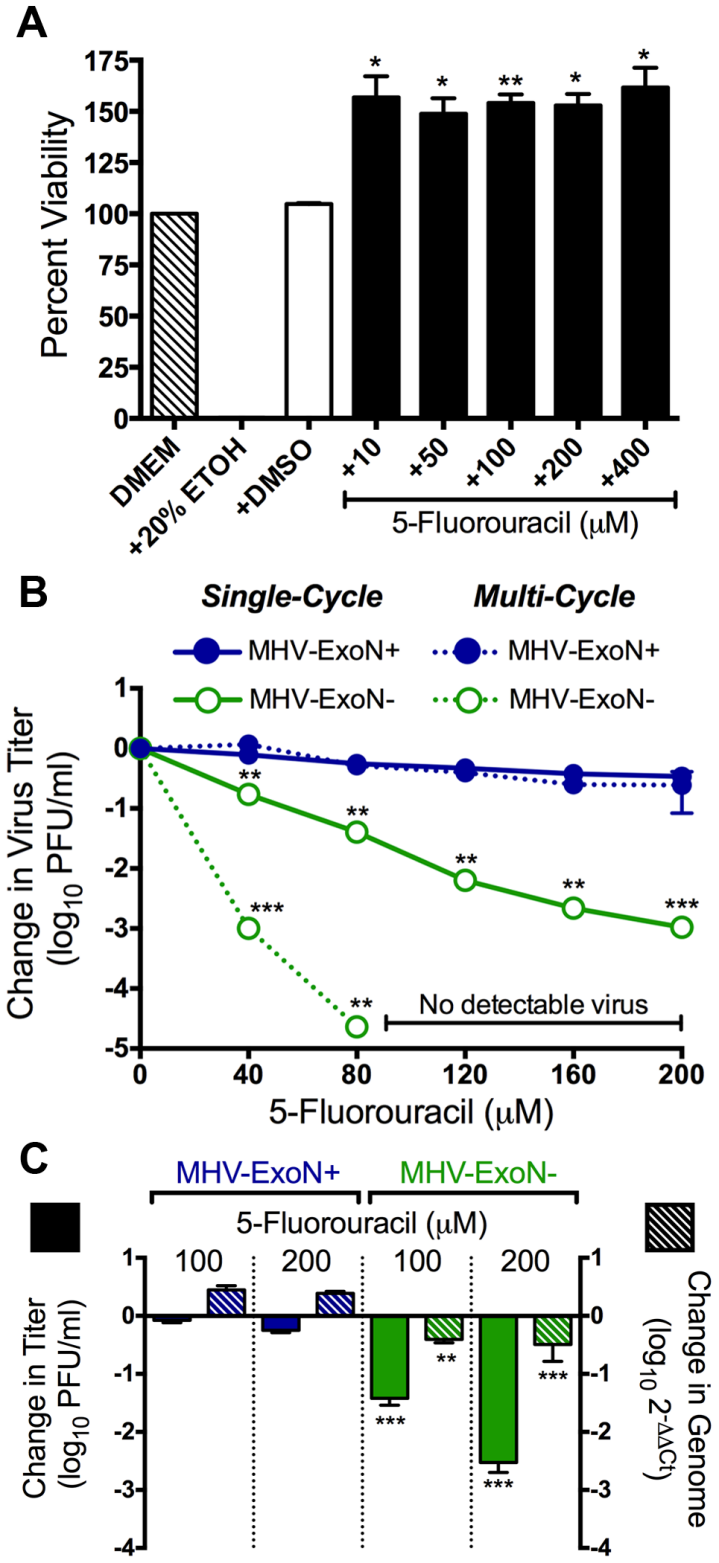
The increased sensitivity of MHV-ExoN− viruses to 5-FU is consistent with mutagenesis. (**A**) DBT cells in 96-well plates were incubated with DMEM alone, or DMEM containing 20% ethanol (EtOH), 4% DMSO, or the indicated concentration of 5-FU for 12 h. Cell viability was determined using CellTiter-Glo (Promega) according to manufacturer's instructions. All values were normalized to the untreated (DMEM) control. Mean values ± S.E.M. are shown, n = 2. (**B**) MHV-ExoN+ (filled circle) and MHV-ExoN− (open circle) virus sensitivity to 5-FU during single- (solid lines; MOI = 1 PFU/cell) and multi-cycle (dotted lines; MOI = 0.01 PFU/cell) replication. MHV-ExoN+ viruses are shown in blue and MHV-ExoN− viruses are shown in green. The change in virus titer was calculated by dividing virus titers following treatment by the untreated controls. Mean values ± S.E.M. are shown, n = 4. (**C**) The change in titer (filled bars) and genomic RNA levels (hatched bars) of MHV-ExoN+ (blue) and MHV-ExoN− (green) viruses following treatment with 5-FU is shown. DBT cells were infected with MHV-ExoN+ or MHV-ExoN− in the presence or absence of 5-FU, and virus titer was determined by plaque assay. Genomic RNA levels were determined using two-step real-time qRT-PCR and primers optimized to amplify a ∼120 nt region of ORF1a [33]. The change in genomic RNA levels (2^−ΔΔCt^) is shown relative to endogenous GAPDH expression and was normalized to RNA levels from untreated samples. Mean values ± S.E.M. are shown, n = 6. For all parts, statistical significance was determined using an unpaired, two-tailed Student's *t* test (*P<0.05, **P<0.01, ***P<0.0001).

Relative specific infectivity values were calculated using the data shown in Figures 1C and 2C and represent the change in virus titer divided by the change in virus genome for each sample. All values are shown relative to untreated virus. The mean value and standard error for each sample is shown (Student's *t* test, n = 4, *P<0.05, **P<0.01, ***P<0.0001).

In addition to decreasing viral RNA synthesis, RBV could be exerting antiviral activity against MHV-ExoN− through competitive inhibition of IMPDH by RMP [51]. To test this possible mechanism, we treated MHV-ExoN+ and MHV-ExoN− viruses with the specific IMPDH inhibitor mycophenolic acid (MPA; [52]–[54]) during both single- and multi-cycle replication. A concentration-dependent decrease in MHV-ExoN− virus titer was observed following MPA treatment during single-cycle replication (Figure 1D). MHV-ExoN+ titers were reduced by less than 10-fold, consistent with what was observed following RBV treatment (Figure 1B). Similar to RBV, increased sensitivity of MHV-ExoN− viruses to MPA was not observed during multi-cycle replication. If RBV is acting via IMDPH inhibition, addition of extracellular guanosine (GUA) should restore virus titers, as has been demonstrated previously for Dengue virus [55]. Addition of 100 µM GUA following RBV or MPA pretreatment and viral infection had no effect on MHV-ExoN+ viruses (Figure 1E), but completely restored MHV-ExoN− titer even in the continued presence of 10 µM RBV or 1 µM MPA (Figure 1F). These data indicate that the antiviral activity of RBV against MHV-ExoN− viruses is occurring at least in part through decreasing viral RNA synthesis and inhibition of IMPDH. Because our primary goal was to test the role of nsp14-ExoN in the prevention and/or removal of nucleotide misincorporation we did not further investigate how RBV was specifically inhibiting ExoN− viruses. However, these results do show that the presence of ExoN activity is capable of preventing RBV inhibition of CoV replication.

### The increased sensitivity of MHV-ExoN− viruses to 5-FU treatment is consistent with mutagenesis

We next examined the sensitivity of MHV-ExoN+ and ExoN− viruses to the pyrimidine base analog 5-FU, which has been shown to be mutagenic for many RNA viruses [29], [56]. Treatment of DBT cells with up to 400 µM 5-FU did not result in any detectable cellular toxicity (Figure 2A). Following treatment with up to 200 µM 5-FU (Figure 2B) during single-cycle infections, MHV-ExoN+ titers were inhibited less than 3-fold, while titers of MHV-ExoN− decreased ∼900 fold, representing a ∼300-fold increase in sensitivity as compared to MHV-ExoN+. During multi-cycle replication, MHV-ExoN+ virus titers were reduced by less than 10-fold following 5-FU treatment, while MHV-ExoN− showed a ∼50,000-fold reduction in titer (Figure 2B). Virus was undetectable by plaque assay at 5-FU concentrations above 80 µM. Analysis of viral RNA synthesis by two-step real-time qRT-PCR demonstrated that MHV-ExoN+ RNA levels were not reduced following 5-FU treatment, while 5-FU treatment resulted in minimal two-to-five fold decreases in MHV-ExoN− RNA (Figure 2C). The specific infectivity of MHV-ExoN− was decreased by 14- and 128-fold following treatment with 100 µM and 200 µM 5-FU, respectively (Table 1). These results demonstrate that ExoN activity confers resistance to 5-FU, and support the hypothesis that 5-FU is driving increased genomic mutagenesis in MHV-ExoN− virus populations, leading to lethal mutagenesis and extinction.

### SARS-ExoN− viruses are sensitive to 5-FU treatment

**Figure 3:**
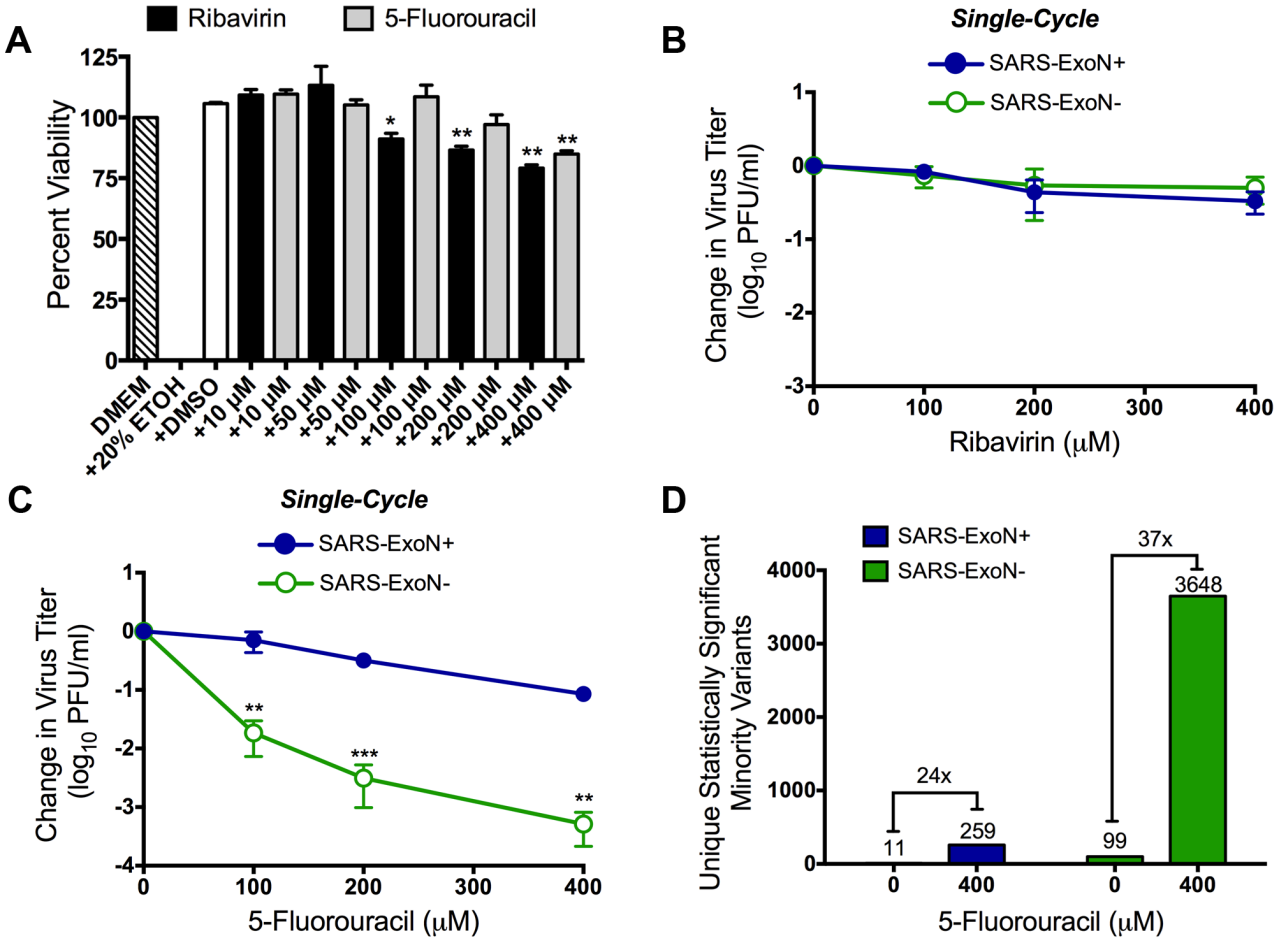
SARS-ExoN− viruses have increased sensitivity to 5-FU. (**A**) Vero cells in 96-well plates were incubated with DMEM alone, or DMEM containing 20% ethanol (EtOH), 4% DMSO, or the indicated concentration of RBV or 5-FU for 24 h. Cell viability was determined using CellTiter-Glo (Promega) according to manufacturer's instructions. All values were normalized to the untreated (DMEM) control. Mean values ± S.E.M. are shown, n = 3. The change in SARS-ExoN+ (filled blue circles) and SARS-ExoN− (empty green circles) titers following treatment with RBV (**B**) or 5-FU (**C**) during single-cycle replication. Vero cells were infected with either virus at an MOI of 0.1 PFU/cell, and virus supernatant was harvest 24 h post-infection following replication in the presence or absence of RBV or 5-FU. Virus titer was determined by plaque assay on Vero cells. Mean values ± S.E.M. are shown, n = 2 (RBV) and n = 4 (5-FU). (**D**) Comparison of unique statistically significant (P<0.05) minority variants present between untreated and 5-FU treated samples for both SARS-ExoN+ and ExoN− populations. SARS-ExoN+ viruses are shown in blue, and SARS-ExoN− viruses are shown in green. For panels A–C statistical significance was determined using an unpaired, two-tailed Student's *t* test (*P<0.05, **P<0.01, ***P<0.0001).

To determine whether SARS-CoV viruses lacking ExoN activity (SARS-ExoN−) also were inhibited by RBV and 5-FU, we infected Vero cells with either SARS-ExoN+ or ExoN− viruses in the presence or absence of RBV or 5-FU. Treatment of Vero cells with up to 400 µM RBV or 5-FU did not decrease cell viability by more than 20% (Figure 3A). Recent reports have described the lack of RBV uptake by Vero cells due to the absence of specific equilibrative nucleoside transporters [57], [58]. Additionally, previous studies have shown that RBV failed to inhibit SARS-CoV replication in Vero cells [59]. Consistent with those reports, in our experiments both SARS-ExoN+ and ExoN− viruses were unaffected by treatment with up to 400 µM RBV (Figure 3B). We therefore performed subsequent experiments with 5-FU. SARS-ExoN+ titers were reduced 3- and 10-fold following treatment with 200 or 400 µM 5-FU, respectively (Figure 3C). In contrast, SARS-ExoN− titers were reduced ∼300-fold by 200 µM 5-FU (Figure 3C), similar to MHV-ExoN− viruses. At 400 µM 5-FU, SARS-ExoN− virus was inhibited 2,000-fold during a single replication cycle, representing a ∼160-fold increase in 5-FU sensitivity compared to SARS-ExoN+ viruses. Thus, our data indicate that increased sensitivity of CoVs to RNA mutagens in the absence of ExoN activity is conserved across diverse members of the CoV family. Of interest, our studies with SARS-ExoN+ also indicate that ExoN-mediated protection from nucleotide misincorporation can be overcome at higher concentrations of mutagen.

### 5-FU drives increased mutagenesis in both SARS-ExoN+ and ExoN− viruses

Studies with the RNA viruses lymphocytic choriomeningitis virus (LCMV), foot-and-mouth disease virus (FMDV) and vesicular stomatitis virus (VSV) have demonstrated that 5-FU is incorporated as 5-fluorouridine monophosphate (FUMP) into replicating viral RNA, thus increasing genomic mutations [60]–[62]. To determine whether 5-FU was causing increased mutagenesis in SARS-CoV populations, we performed full-genome NGS analysis of both virus populations replicating in the presence or absence of 5-FU. To analyze the entire spectrum of mutations arising during replication, we extracted total intracellular RNA from Vero cells infected with either SARS-ExoN+ or ExoN− viruses following treatment with either 0 µM or 400 µM 5-FU. We then generated 12 overlapping cDNA amplicons of approximately 3 kb in length for each sample. For each of the four samples, 1.4×10^8^ to 4.5×10^8^ bases were sequenced, corresponding to an average coverage depth of between 4,600 and 15,000 at each nucleotide position. We compared the statistically significant minority variants, defined as having a p-value of ≤0.05 following a multiple-testing correction (Benjamini-Hochberg), between the untreated and 5-FU-treated SARS-ExoN+ and ExoN− populations. Following treatment with 400 µM 5-FU (Figure 3D), there was an increase in mutations within the SARS-ExoN+ population from 11 to 259 (24-fold). In contrast, for SARS-ExoN− there were 3648 mutations present within the 5-FU-treated SARS-ExoN− population compared to the 99 mutations in the untreated population (40-fold increase). Most remarkably, this represented a 16-fold increase in the number of statistically significant minority variants between 5-FU treated ExoN+ and ExoN− SARS-CoV. Thus, these data support our hypothesis that 5-FU was increasing genomic mutations through incorporation of FUMP into viral genomes in the absence of ExoN activity.

### 5-FU-associated A-to-G and U-to-C transitions are highly represented and distributed across the genome

**Figure 4:**
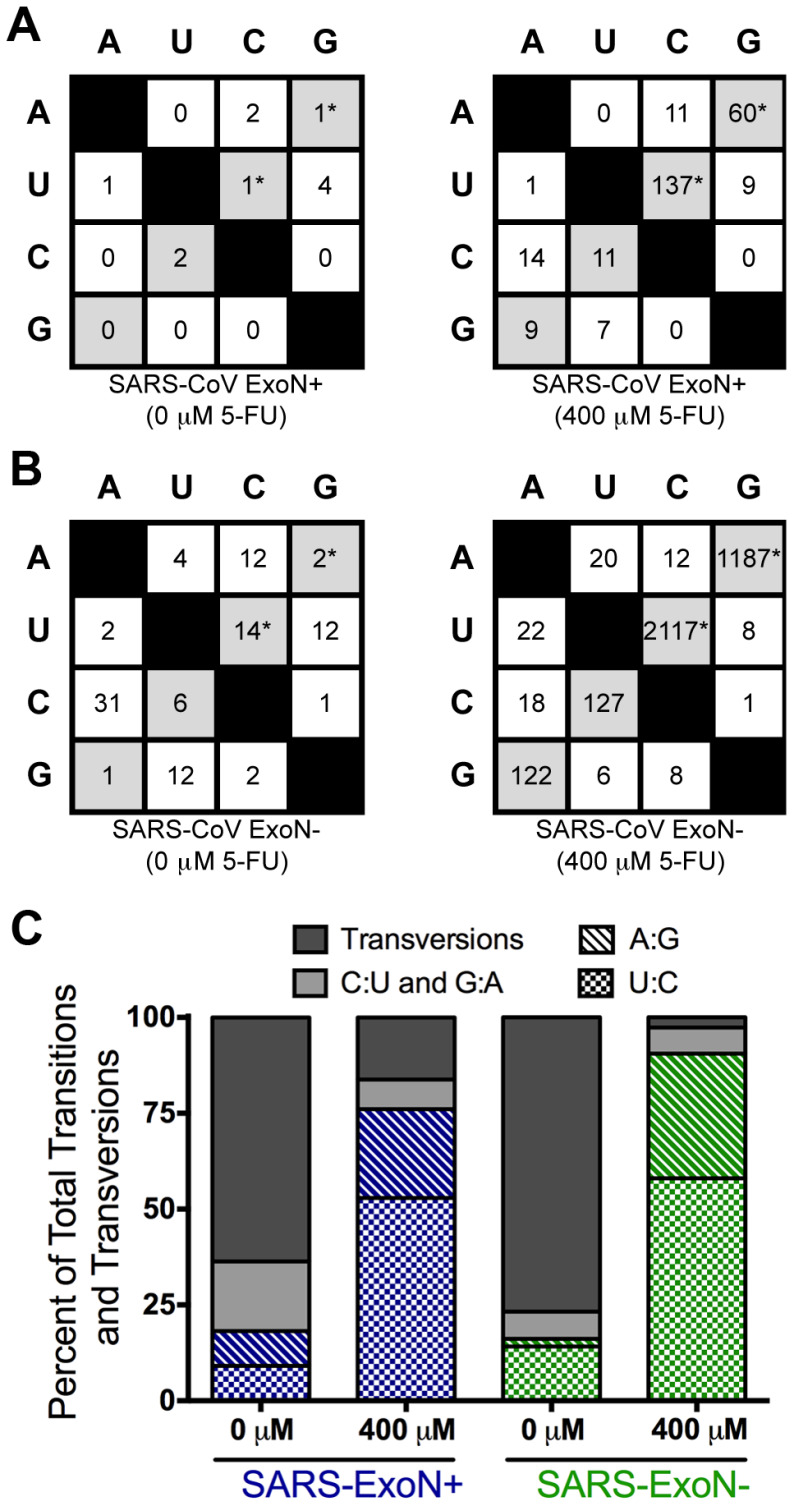
Incorporation of FUMP results in increased U:C and A:G transitions. All possible base changes are shown for SARS-ExoN+ and SARS-ExoN− viruses in panels (**A**) and (**B**), respectively. Transitions (A↔G and U↔C) are shaded in grey, and 5-FU specific transitions (U:C and A:G) are marked with an asterisk. Transversions (A↔T, A↔C, C↔G, G↔T) are shown in white boxes. All values represent the number of unique statistically significant minority variants following 5-FU treatment. (**C**) The percent of all unique statistically significant minority variants represented by transversions (filled dark grey bars), C:U and G:A transitions (filled light grey bars), and the 5-FU specific transitions A:G (hatched bars) and U:C (checkered bars) are shown following 0 or 400 µM 5-FU treatment. SARS-ExoN+ viruses are shown in blue, and SARS-ExoN− viruses are shown in green.

Incorporation of FUMP instead of uracil into replicating RNA allows FUMP to base pair with both guanosine and adenine [61], [63]. This decreased specificity in base pairing has been shown in studies with LCMV and primarily results in A-to-G (A:G) and U-to-C (U:C) transitions [29], [61], [63]. To determine if FUMP was being incorporated at higher levels in the absence of ExoN-mediated proofreading, we analyzed the numbers and types of transitions and transversions occurring in each virus population (Figure 4). Transitions are indicated in grey boxes and transversions in white boxes, with the number for each shown. Transversions comprised the majority of variants for both untreated ExoN− and ExoN+ viruses. Treatment with 5-FU caused the number of U:C and A:G transitions to increase in both ExoN+ and ExoN− populations, from 2 to 197 for SARS-ExoN+ and from 16 to 3304 for SARS-ExoN− (Figures 4A and B). This increase and bias toward U:C and A:G transitions is consistent with FUMP being incorporated into both minus- and plus-strand RNA [63] during both ExoN+ and ExoN− replication; however the absolute numbers were dramatically increased (16-fold) during ExoN− replication compared to ExoN+. In untreated cells, A:G and U:C transitions accounted for less than 25% of the total minority variants within each population (Figure 4C). Following 5-FU treatment, A:G and U:C transitions accounted for 70–95% of the total minority variants within each population.

**Figure 5:**
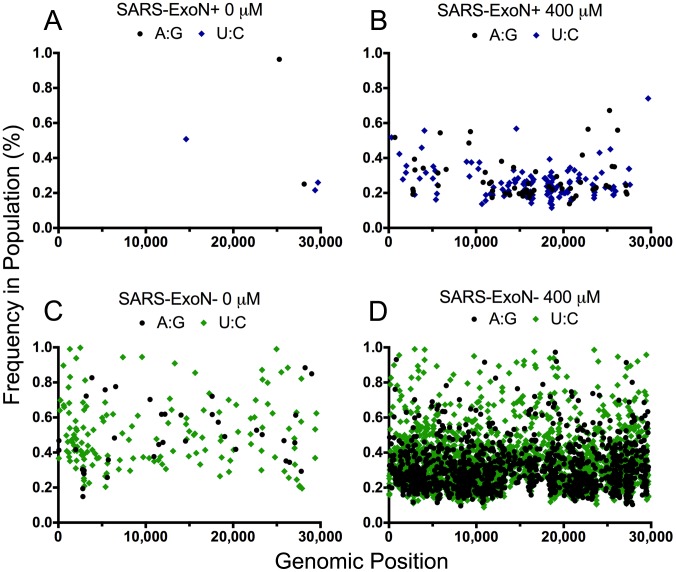
5-FU-mediated U:C and A:G transitions are distributed across the CoV genome at low frequency. (**A**) and (**B**) The genomic distribution of low frequency statistically significant U:C and A:G variants within the SARS-ExoN+ population following treatment with 0 or 400 µM 5-FU. (**C**) and (**D**) Same as in A and B except for the SARS-ExoN− population. For all panels, SARS-ExoN+ viruses are shown in blue, and SARS-ExoN− viruses are shown in green. U:C transitions are denoted by a diamond, whereas A:G transitions are plotted as circles.

To further examine the genomic distribution of these two transitions, we plotted the total number of A:G and U:C transitions occurring at a frequency of between 0.1% and 1% (Figure 5). Approximately 75% and 90% of the total minority variants occurring at a frequency between 0.1 and 1% following 5-FU treatment were due to A:G or U:C transitions (Figure 5), for the SARS-ExoN+ and ExoN− populations, respectively. In both populations, these mutations were distributed across the entire genome following treatment with 400 µM 5-FU. Thus our data provide direct evidence indicating that 5-FU drives increased genomic mutations within SARS-CoV in the absence of ExoN proofreading activity.

## Discussion

Viral sensitivity to RNA mutagens is determined by several factors including polymerase selectivity [39], [40], [64]–[67], mutational robustness [68], and the acquisition of mutations that increase or decrease replication fidelity. Increased and decreased fidelity mutants have been described for picornaviruses and arboviruses [35], [48], [50], [69], all of which have occurred in the viral RdRp. The CoV nsp14-ExoN is the first identified RNA virus protein distinct from the RdRp that affects replication fidelity [19]–[21], [70]. While the G641D mutation within the chikungunya (CHIKV) nonstructural protein 2 (nsP2) has been implicated in CHIKV resistance to RBV, a direct role for this protein in fidelity regulation has not been described [48]. A Sindbis virus variant containing mutations within nsP1, a viral guanylyl- and methyltransferase [71], has been shown to be resistant to both RBV and MPA [72]. However, this phenotype is related to viral RNA capping and not replication fidelity [72]. In this report, we identify CoV ExoN activity as a critical determinant of viral sensitivity to RNA mutagens. Using two phylogenetically distant β-CoVs we demonstrate that this phenotype is well conserved across CoVs. Clearly, there is a profound increase both in overall mutations and in specific 5-FU-associated mutations within the ExoN− population as compared to the ExoN+ wild-type population. Furthermore, the vast majority of statistically significant mutations were distributed genome-wide at frequencies between 0.2 and 1%, providing strong evidence supporting ExoN-mediated proofreading during CoV replication. Of interest, our experiments also revealed that ExoN-mediated prevention and/or removal of misincorporated nucleotides is not absolute, especially in the setting of higher concentrations of mutagen. ExoN+ SARS-CoV populations demonstrated 24-fold more mutations following 5-FU treatment, suggesting that ExoN proofreading can be overwhelmed by higher concentrations of mutagens and likely by other nucleoside or base analogs. This raises the further possibility that ExoN may be less efficient at recognizing or removing some types of nucleoside or base analogs than others, and that such approaches to virus inhibition might be viable, particularly in combination with inhibitors that target ExoN activity.

### Ribavirin activity against CoVs is not primarily due to mutagenesis

The antiviral nucleoside analog RBV is currently used to treat hepatitis C virus (HCV; [73]–[75]), Lassa virus [76] and respiratory syncytial virus (RSV) infections [77], [78]. The potential clinical use of RBV for CoV infections is complicated by the multiple mechanisms of action that have been reported [38], and by the potential for disease exacerbation, as reported during the SARS-CoV epidemic [25]–[28]. Our data suggest that RBV primarily inhibits MHV-ExoN− virus replication through decreasing viral RNA synthesis and inhibition of IMPDH (Figure 1). Inhibition of IMPDH by RMP has been shown to decrease intracellular GTP pools [51], thus altering the balance of nucleoside triphosphates (NTPs) within the cell. Decreased GTP levels could result in forced misincorporations due to NTP imbalances in the absence of ExoN activity [72]. However, the moderate 6- to 9-fold decreases in relative specific infectivity observed for MHV-ExoN− following RBV treatment (Table 1) suggests that mutagenesis is not the primary mechanism by which RBV is exerting an antiviral effect. An additional possibility is that the antiviral activity of RBV against ExoN− viruses is unrelated to the putative proofreading function of this enzyme. Both biochemical and cell culture studies have demonstrated that loss of ExoN activity leads to impaired RNA synthesis [15], [19], [20]. Furthermore, in addition to ExoN activity, nsp14 contains N7-methyltransferase (N7-MTase) activity, a critical step in RNA capping [79], [80]. A recent report has demonstrated that the ExoN and N7-MTase domains are structurally inseparable, and that residues within the ExoN domain are important for N7-MTase activity [81]. Thus, the increased sensitivity of MHV-ExoN− to RBV could result from the impairment of undefined functions of ExoN during CoV replication, particularly during RNA synthesis. The parallel use of ExoN+ and ExoN− viruses with RBV may allow us to define how RBV is exerting an antiviral effect against CoVs and the potentially novel mechanisms by which ExoN may act to counter that inhibition.

### ExoN proofreading during CoV replication

Since the identification of nsp14-ExoN activity [15] and studies demonstrating the requirement for ExoN in high-fidelity replication [19]–[21], mounting evidence points to a role for nsp14-ExoN in proofreading activity during RNA virus replication [22]. Here we used NGS to determine the number of mutations present in SARS-ExoN+ and ExoN− populations. The characteristic 5-FU-mediated transitions U:C and A:G comprised 90% of the total statistically significant minority variants within SARS-ExoN− population, and were present at levels 15- and 20-fold higher than those same transitions within the ExoN+ population (Figure 4). Overall, our data represent the first direct test of ExoN proofreading during SARS-CoV replication in the absence of ExoN. Furthermore, the sequencing depth attained using NGS shows that ExoN inactivation likely skews the spectrum of spontaneous mutations present within the untreated population (Figure 4). Such overrepresentation of specific mutations in the context of ExoN inactivation is similar to studies of *S. cerevisiae* DNA polymerases ε and δ containing mutations within their respective 3′-to-5′ DEDD exonucleases [82]–[86]. This altered distribution due to ExoN inactivation could have profound implications for CoV adaptation and evolution.

### Nsp14-ExoN as a target for combination CoV inhibitors

Lethal mutagenesis occurs through the accumulation of mutations within the viral genome during replication, and ultimately results in virus extinction (reviewed in [56], [87]). While lethal mutagenesis has been studied extensively [87], our work is the first to identify an RNA virus protein distinct from the RdRp that directly regulates the sensitivity of RNA viruses to genomic mutations resulting from mutagen incorporation. Currently, RBV is the only FDA-approved antiviral with demonstrated mutagenic activity. The first demonstration of RBV acting as a mutagen was performed using poliovirus [23], [24] almost 30 years after the antiviral activity of RBV was described [88]. The nucleoside analog T-705 (Favipiravir; [89]) is currently in clinical development, and has been shown recently to drive lethal mutagenesis of influenza virus [90]. We have shown that ExoN+ viruses replicate well in the presence of RBV or 5-FU. However, we also have shown that ExoN− mutants of SARS-CoV and MHV have 15-to-20-fold decreased fidelity [19], [20], are attenuated, are subject to rapid loss of replication and clearance *in vivo* [21], and are highly susceptible to low concentrations of RNA mutagens. An exciting possibility is that this conserved CoV proofreading enzyme could be targeted for inhibition, thus leading to the development of broadly useful CoV therapeutics. While ExoN inhibitors alone might be efficacious, combining an inhibitor of CoV fidelity with an RNA mutagen would magnify the intrinsic fidelity defect of ExoN inhibition and drive high-level mutagenesis. A potential advantage of such an approach would be to rapidly drive the virus to extinction, while limiting or blocking the capacity of the virus to overcome inhibition by reversion. ExoN− mutants of both MHV and SARS-CoV have shown no reversion over multiple passages in culture or during persistent infections *in vivo* [19]–[21]. Furthermore, we did not observe any primary reversions within the ExoN DEDD motif following 5-FU treatment. While mutations within the CoV RdRp could emerge during acute treatment, mutations within other RNA virus RdRps have demonstrated that the maximum tolerance for increased or decreased fidelity without loss of virus viability is between ∼3- to 6-fold [35], [48], [69], [91]. In addition, our data demonstrate that ExoN− viruses are profoundly sensitive to inhibition by lower concentrations of mutagen, providing a possible improved therapeutic index and margin of safety for use.

In summary, this study provides the most direct evidence to date that CoV ExoN provides a proofreading function during virus replication, and identifies ExoN as the critical determinant of CoV sensitivity to RNA mutagens. Because CoV replication fidelity is likely determined by the concerted effort of multiple virus proteins [19], [20], [22], our data suggest the exciting possibility that significant attenuation of CoV fitness and pathogenesis could be achieved by targeting the conserved process of CoV replication fidelity. Ultimately, uncovering the mechanism of fidelity regulation and methodologies to disrupt this critical process will be vital to responding to both endemic and future emerging CoVs such as SARS-CoV and MERS-CoV.
