# Correction: Coronaviruses Lacking Exoribonuclease Activity Are Susceptible to Lethal Mutagenesis: Evidence for Proofreading and Potential Therapeutics

**DOI:** 10.1371/journal.ppat.1004342

**Published:** 2014-07-30

**Authors:** The PLOS Pathogens Staff

Matthew C. Surdel is not included in the author byline. He should be listed as the third author and affiliated with the Department of Pathology, Microbiology and Immunology, Vanderbilt University School of Medicine, Nashville, Tennessee, United States of America. The contribution of the author is as follows: He performed initial experiments demonstrating effects of mutagens on ExoN- mutant viruses.

The following information is missing from the funding section: Support for MS was provided by T32 GM007347 from the National Institute of General Medical Studies for the Vanderbilt Medical-Scientist Training Program.
